# *Aspergillus terreus* and the Interplay with Amphotericin B: from Resistance to Tolerance?

**DOI:** 10.1128/aac.02274-21

**Published:** 2022-03-07

**Authors:** Roya Vahedi-Shahandashti, Anna-Maria Dietl, Ulrike Binder, Markus Nagl, Reinhard Würzner, Cornelia Lass-Flörl

**Affiliations:** a Institute of Hygiene and Medical Microbiology, Medical University of Innsbruck, Innsbruck, Austria

**Keywords:** *Aspergillus terreus*, tolerance, amphotericin B, antifungal susceptibility testing, amphotericin B tolerance, aspergillosis, antifungal resistance, clinical breakpoint

## Abstract

Aspergillus terreus is an opportunistic causative agent of invasive aspergillosis and, in most cases, it is refractory to amphotericin B (AMB) therapy. Notably, AMB-susceptible Aspergillus terreus *sensu stricto* (s.s.) representatives exist which are also associated with poor clinical outcomes. Such findings may be attributable to drug tolerance, which is not detectable by antifungal susceptibility testing. Here, we tested *in vitro* antifungal susceptibility (AFST) and the fungicidal activity of AMB against 100 clinical isolates of A. terreus species complex in RPMI 1640 and antibiotic medium 3 (AM3). MICs ranged from 0.5 to 16 μg/mL for RPMI 1640 and from 1 to >16 mg/L for AM3. AMB showed medium-dependent activity, with fungicidal effects only in antibiotic medium 3, not in RPMI 1640. Furthermore, the presence of AMB-tolerant phenotypes of A. terreus has been examined by assessing the minimum duration for killing 99% of the population (MDK99) and evaluating the data obtained in a Galleria mellonella infection model. A time-kill curve analysis revealed that A. terreus with AMB MICs of ≤1 mg/L (susceptible range) displayed AMB-tolerant phenotypes, exhibiting MDK99s at 18 and 36 h, respectively. Survival rates of infected G. mellonella highlighted that AMB was effective against susceptible A. terreus isolates, but not against tolerant or resistant isolates. Our analysis reveals that A. terreus isolates which are defined as susceptible based on MIC may comprise tolerant phenotypes, which may, in turn, explain the worse outcome of AMB therapy for phenotypically susceptible isolates.

## INTRODUCTION

Invasive fungal infections (IFIs) are a leading cause of infectious morbidity and mortality (1, 2). Species belonging to Aspergillus, the most common opportunistic mold representatives, cause an array of superficial to deep-seated systemic infections (3, 4). Their immunological status triggers the onset of infection and clinical manifestations (5, 6). Antifungal treatment remains a challenge (7) and, currently, only four classes of antifungal agents are available in clinical routines to treat IFIs; namely, azoles, polyenes, echinocandins, and 5-flucytosine (8). Amphotericin B (AMB) remains the broadest drug available, with only a few fungal pathogens harboring primary or acquired resistance; essentially, the rising number of fungi with reduced azole-susceptibility is a matter of concern (9–12).

Although A. fumigatus represents the most prevalent species involved in invasive aspergillosis (13), members of the sections *Terrei* or *Flavi* are the second or third most important species in specific regions (14–16). A current multicenter study surveying the global prevalence of A. terreus species complex demonstrated an overall occurrence of 5.2% (among 370 cases of fungal disease) (17). A. terreus holds an exceptional clinical state by representing a high propensity for dissemination and mortality (51 to 70%) in invasive aspergillosis (14, 18–21). Most A. terreus isolates display high (>2 mg/L) AMB MICs, and hence have been considered intrinsically resistant (22–25). However, some studies have reported A. terreus isolates showing a wide range of AMB MICs, including low MICs (<0.5 mg/L) (26–28). Interestingly, high virulence potential was observed both in a mouse model and in a Galleria mellonella model infected with AMB-susceptible strains (24, 29, 30). The broad ranges of AMB-MIC phenotypes and MIC-independent clinical outcomes (31, 32) led us to investigate the role of tolerance in A. terreus under miscellaneous growth conditions (33–36). This study analyzed the efficacy of AMB against isolates of section *Terrei* by (i) evaluating MIC ranges in RPMI 1640 and antibiotic medium 3 (AM3), (ii) determining minimum fungicidal concentrations (MFCs), (iii) examining AMB killing-kinetic patterns to define tolerant phenotypes, (iv) evaluating germination rates, and finally (v) assessing AMB efficacy in the G. mellonella infection model.

## RESULTS

### Medium-dependent shift in AMB MIC distribution.

The MIC distributions for AMB against 100 clinical isolates of Aspergillus section *Terrei*, which provided a sufficiently large sample size, have been determined for RPMI 1640 and AM3, and range from 0.5 to 16 mg/L and from 1 to >16 mg/L, respectively (Table 1). The MIC_50_ and MIC_90_ were 2 and 4 mg/L for RPMI 1640, shifting to 8 and 16 mg/L for AM3 (Table 1).

**TABLE 1:** Susceptibility profiles of amphotericin B against Aspergillus section *Terrei* in RPMI 1640 and AM3^*a*^

Species (no. of isolates)	RPMI 1640						AM3					
	MIC (mg/L)^*b*^			MFC (mg/L)			MIC (mg/L)			MFC (mg/L)		
	Range	MIC_50_	MIC_90_	Range	MFC_50_	MFC_90_	Range	MIC_50_	MIC_90_	Range	MFC_50_	MFC_90_
*A.* section *Terrei* (100)^*c*^	0.5−16	2	4	>16	>16	>16	1−>16	8	16	4−>16	16	>16
A. terreus s.s. (78)	0.5-4	2	4	>16	>16	>16	1−>16	8	16	4−>16	16	>16
*A. hortai* (10)	0.5−2	1	2	>16	>16	>16	4−16	8	16	4−>16	16	>16
*A. citrinoterreus* (11)	1−16	2	8	>16	>16	>16	4−16	8	16	4−>16	16	>16
*A. alabamensis* (1)	1			>16			8			8		

a AM3, antibiotic medium 3; MFC, minimum fungicidal concentration; s.s., *sensu stricto*.

b MIC_50_, MIC_90_, MFC_50_, and MFC_90_ are only shown for species with 10 or more isolates.

c Isolate S164 (susceptible control) is not included in this table.

### AMB showed medium-dependent fungicidal activity.

AMB did not exhibit fungicidal activity against tested isolates (*n* = 100) in RPMI 1640; all fungi displayed MFCs of >16 mg/L (Table 1). In contrast, in AM3, AMB showed a different fungicidal profile, with MFCs ranging from 4 to >16 mg/L.

### AMB tolerant phenotypes required a longer time to be killed than the susceptible representative.

**FIG 1:**
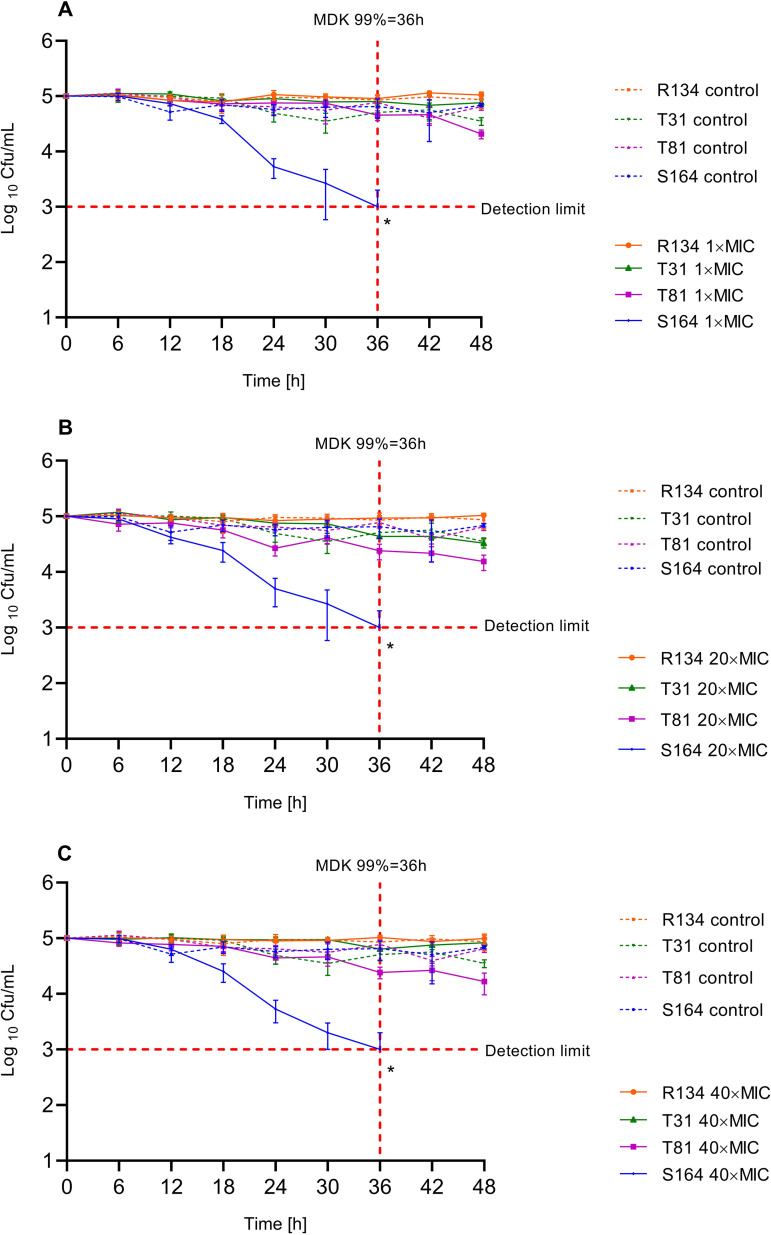
Time-kill kinetics of selected A. terreus isolates following exposure to different concentrations of amphotericin B at 1× (A), 20× (B), and 40× MIC (C) in RPMI 1640 medium supplemented with 2% glucose. Red horizontal dotted lines indicate limit of detection (99% of the initial population). Red vertical dotted lines show minimum duration of killing times needed to kill 99% of the initial inoculum (MDK99). All data represent mean values of three independent experiments (*, *P* < 0.05). For clarity, plots illustrate only the statistical significance of the first time points which reached the detection limit.

In RPMI 1640, T81, T31, and R134 did not reach killing detection limits at any concentrations over 48 h, indicating a lack of tolerant phenotypes. The susceptible control (S164) reached the 99%-killing detection limit after 36 h exposure to AMB, irrespective of concentration (*P* = 0.0005) (Fig. 1A to C). Due to the fungicidal activity of AMB even at low concentrations (1× MIC), S164 is considered to be susceptible.

**FIG 2:**
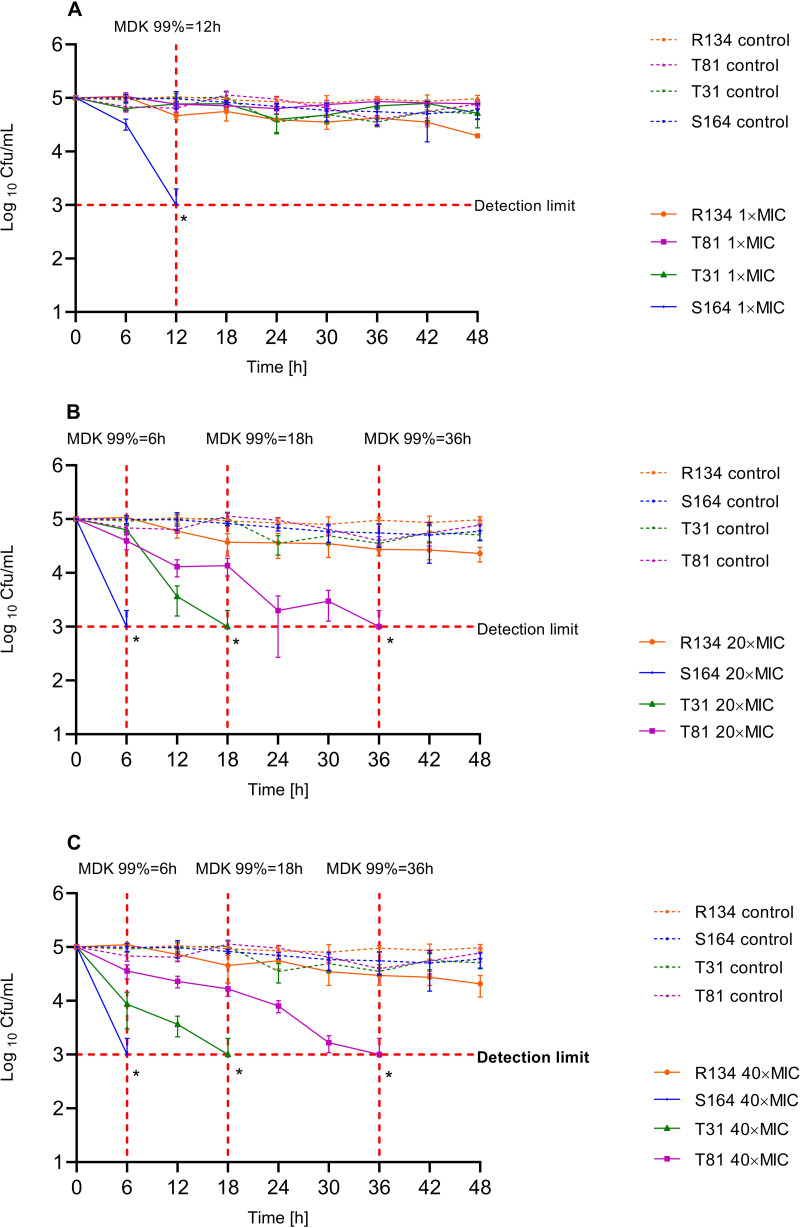
Time-kill kinetics of selected A. terreus isolates following exposure to different concentrations of amphotericin B at 1× (A), 20× (B), and 40× MIC (C) in antibiotic medium 3 (AM3). Red horizontal dotted lines indicate limit of detection (99% of the initial population). Red vertical dotted lines indicate minimum duration of killing time needed to kill 99% of the initial inoculum (MDK99). All data represent mean values of three independent experiments (*, *P* < 0.05). For clarity, plots illustrate only the statistical significance of the first time points which reached the detection limit.

In contrast, time-kill curves in AM3 showed different AMB-killing patterns (Fig. 2). AMB concentrations of 1× MIC (Fig. 2A) resulted in a faster and shorter MDK99 of 12 h (*P* < 0.0001) for S164 compared to other isolates, which failed to reach the 99%-killing detection limit within 48 h at 1× MIC. In addition, S164 showed time- and concentration-dependence, with a MDK99 of 6h at 20× (*P* < 0.0001) and 40× MIC (*P* < 0.0001). T81 and T31 showed tolerant phenotypes at 20× (*P* = 0.0101 and *P* < 0.0001, respectively) and 40× MIC (*P* = 0.0125 and *P* < 0.0001, respectively), displaying MDK99s of 36 h and 18 h, respectively (Fig. 2B and C). R134 was shown to be AMB-resistant by not reaching the 99%-killing detection limit in any of the concentrations and time points tested (Fig. 2A to C).

### *A*. *terreus* showed an increased germination rate in AM3 compared to that in RPMI 1640.

All isolates showed significantly higher rates of germination in AM3 compared to those in RPMI 1640. Following 12 h of incubation in AM3, the average germination rates for S164, R134, T81, and T31 were 95.3% (*P* = 0.0059), 86.6% (*P* = 0.0016), 100% (*P* = 0.0023), and 90.3% (*P* = 0.0040), respectively. In contrast, the germination rates were lower in RPMI 1640: 12.6% for S164, 10.6% for R134, 21% for T81, and 20% for T31.

### AMB efficacy in *A*. *terreus*-infected *G*. *mellonella* larvae showed a good correlation with *in vitro* MDK99 compared to MIC.

**FIG 3:**
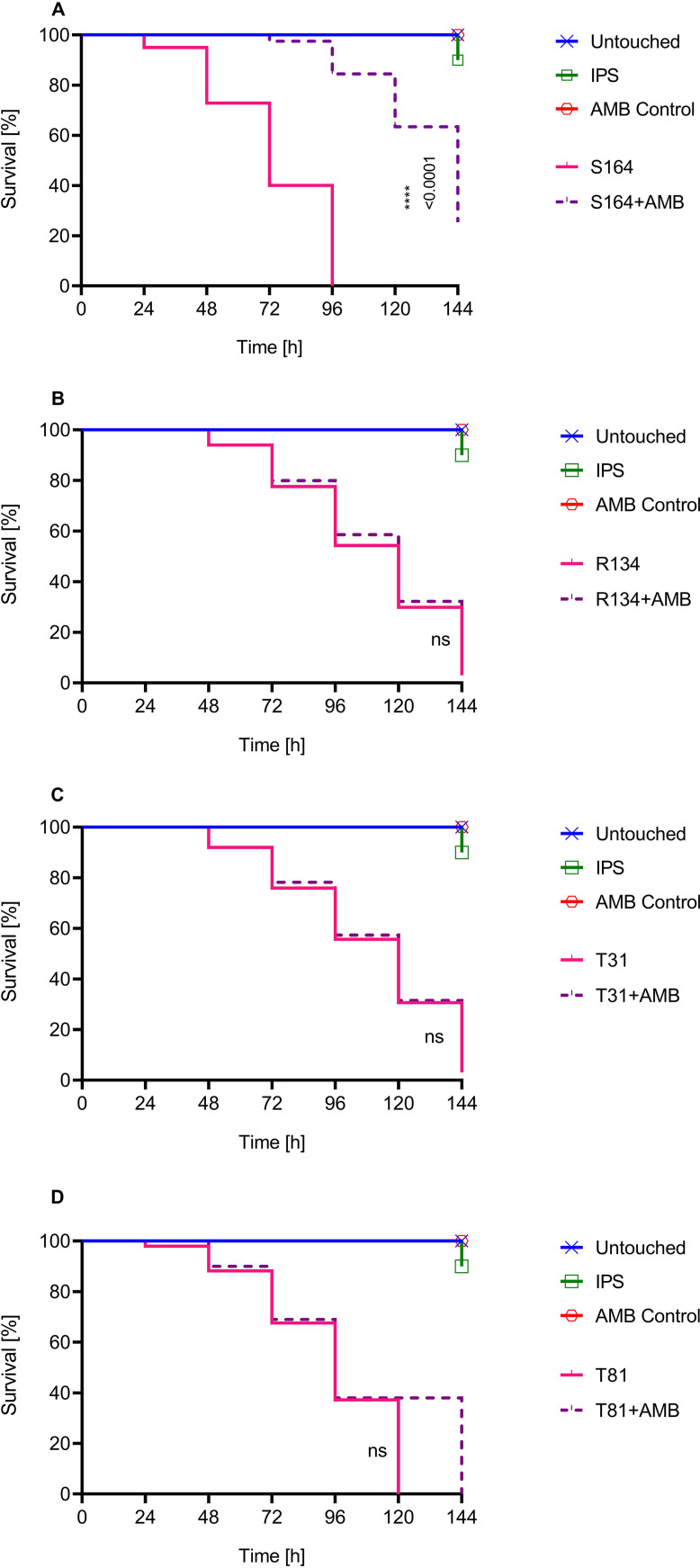
Kaplan-Meier survival curve for G. mellonella larvae following inoculation with different A. terreus isolates: S164 (A), R134 (B), T31 (C), and T81 (D). Larvae were infected with 1 × 10^7^ spores; 2 h later, either amphotericin B (AMB; 0.5 μg per larva) or insect physiological saline (IPS) was administered, and larvae were incubated at 37°C. Survival was monitored every 24 h over 144 h (6 days). Curves represent the average of three independent experiments (60 larvae in total). *P* values for significantly different results are shown (*P* < 0.05; Mantel-Cox test); otherwise, results are marked as not significant (ns).

Treatment with AMB was only successful in larvae infected with S164 (Fig. 3A), as shown by their prolonged survival compared to groups that did not receive AMB (*P* < 0.0001). AMB administration did not influence the survival of larval infected with R134 (Fig. 3B), T31 (Fig. 3C), or T81 (Fig. 3D).

## DISCUSSION

The discordance between MICs in a susceptible range and poor therapy outcomes (31, 37) may be attributable to drug tolerance, which is not detectable by AFST (33, 38, 39). AMB MIC distributions in RPMI 1640 showed a broad range, which is in line with the results of previous studies (17, 36, 40–43); in comparison, MIC_50_ and MIC_90_ shifted to higher concentrations when applying AM3 (Table 1). In alignment with previous studies, AMB did not show any fungicidal activity in RPMI 1640 at any concentration tested (41, 44). In contrast, fungicidal activities of the polyene were observed to utilize AM3 (Table 1). This indicates that for selected A. terreus strains, AMB may act fungistatic in RPMI 1640 and fungicidal in AM3. These findings underline the importance of considering any effects of nutrient media when assessing biomedical tests (34, 45–47).

We hypothesized that a lack of discrimination between AMB-susceptible and AMB-tolerant A. terreus phenotypes in the application of standard AFST could be a reason for treatment failure in patients infected with susceptible isolates (20, 37, 48–50). MIC alone is not an adequate metric for tolerance detection, and relying solely on MICs might lead to suboptimal or inappropriate therapy (33, 38, 39). Tolerance in bacteria or/and yeasts is a multifactorial phenomenon, either associated with a genetic basis (inherited or non-inherited) or conferred by environmental and nutritional factors (33, 38, 51–53). To our knowledge, no study has addressed the detection of tolerance in molds; however, attempts have been made to develop different methods for tolerance detection based on the type of microorganism and the cidal or static activity of the agent (33, 51, 54, 55). Based on recent studies, we aimed to assess AMB tolerance among A. terreus s.s. by applying MDK99 detection (33).

In our study, two strains of A. terreus s.s., with MICs in the susceptible range, demonstrated tolerant phenotypes in AM3, but not in RPMI 1640, according to MDK99 (Fig. 1 and 2). Polyene antifungal activity may be influenced by medium composition (44, 56, 57). Besides this, nutrient-limited media affects fungal growth and, in consequence, tolerance (33, 38). An optimal nutrient environment provides sufficient nutrients to allow molds to grow and germinate without restriction (58). In agreement with the results of the previous study, our results confirmed that utilizing a nutrient-limited media such as RPMI 1640, although supplemented with 2% glucose, causes fungi to germinate at a lower rate than in AM3, which contains complex nutrients (58). This, in turn, might result in deviating results for the fungicidal activity of AMB in nutrient-limited media (34, 35, 54, 56, 58). The use of AM3 resulted in higher germination rates as well as improved detection of the fungicidal activity of AMB. Our data suggest AM3 be superior to RPMI 1640 when evaluating AMB activity; however, further studies are necessary until new recommendations can be made for AFST.

These data support the existence of AMB-tolerant phenotypes in A. terreus s.s. T81 and T31 required longer times (36 and 18 h, respectively) to reach the 99%-killing detection limit in high AMB concentrations (20× and 40× MIC), although they displayed MICs in a susceptible range. Irrespective of the medium, the AMB-susceptible isolate was killed in 1× MIC of AMB, while tolerant isolates survived at this concentration, similar to the resistant isolate, by not reaching the 99%-killing detection limit (Fig. 1A and Fig. 2A). Only tolerant isolates could withstand high concentrations of AMB for a long time (Fig. 2B and C). These findings verify that longer exposure to an agent, rather than a higher concentration, is required to produce the same level of killing in a tolerant strain as in a susceptible one (33, 38). Besides this, the MIC-based resistant isolate could not be killed by AMB, irrespective of medium, concentration, or duration of treatment (Fig. 1 and Fig. 2A–C).

To analyze whether AMB tolerance correlated with the *in vivo* efficacy of AMB, we performed treatment studies utilizing G. mellonella larvae (Fig. 3). Our results showed that AMB administration increased the survival of larvae infected with the susceptible strain (Fig. 3A); this finding correlates with those of previous studies (24, 30). In contrast, AMB treatment did not improve the survival of larvae infected with resistant (Fig. 3B) or tolerant A. terreus representatives (Fig. 3C and D). In line with our results, a recent study (51) showed that fluconazole treatment was not effective in G. mellonella infected with fluconazole-tolerant Candida albicans. Further clinical studies regarding the rate of tolerance and its outcome are lacking.

The results of this study indicate that MDK99s are better predictors of treatment outcome than MICs alone. Tolerant isolates required longer MDK99s to reach the 99%-killing detection limit (Fig. 2A–C) than susceptible ones (Fig. 2A–C); in addition, tolerant phenotypes behaved as resistant isolates in larvae upon AMB treatment. The appearance of an AMB-tolerant phenotype might explain the discordance of MIC values obtained by standardized assays and the lack of AMB efficacy *in vivo*.

In conclusion, we suggest that AMB efficacy is affected by the use of fungal growth medium (36, 45, 46, 58), which results in an increased MIC_50_ and MIC_90_ and in broader MFC ranges in AM3 than in RPMI 1640 when testing 100 clinical isolates of section *Terrei.* We underline the presence of tolerant phenotypes within the A. terreus population, showing MICs of ≤1 mg/L. *In vivo*, tolerant phenotypes responded to AMB similarly to resistant strains, despite showing AMB MICs in a susceptible range. Hence, distinguishing tolerance from susceptibility may be adequate before starting AMB treatment for A. terreus-related infections in selected cases.

## MATERIALS AND METHODS

### Fungal strains, growth conditions, and inoculum preparation.

A total of 100 clinical and sequenced isolates of Aspergillus section *Terrei*, including A. terreus s.s. (*n* = 78), *A. hortai* (*n* = 10), *A. citrinoterreus* (*n* = 11), and *A. alabamensis* (*n* = 1) were analyzed. Strains were collected and previously molecularly classified by the ISHAM-ECMM-EFISG TerrNet Study group (www.isham.org/working-groups/aspergillus-terreus) (10, 17). Isolates were cultured from 10% glycerol frozen stocks (–80°C) on Sabouraud dextrose agar (SDA) (BD, Difco) at 37°C for 3 to 5 days; conidia were harvested by applying spore suspension buffer (0.9% NaCl, 0.01% Tween 20 [Sigma-P1379]). Isolate S164 acts as external susceptible control and has not been included in the 100 clinical isolates panel (30).

### Antifungal agent.

Deoxycholate amphotericin B (Sigma-Aldrich, A2411) was utilized in this study. AMB was dissolved in dimethyl sulfoxide (DMSO; Sigma-Aldrich).

### Antifungal susceptibility testing.

Antifungal susceptibility testing for AMB was carried out according to EUCAST guidelines (www.EUCAST.org) (59), using two different nutrition media, including RPMI 1640 medium (R6504, Sigma) supplemented with 2% glucose buffered to pH 7.0 with 0.165 M morpholinepropanesulfonic acid (MOPS; Sigma), as a standard medium recommended by EUCAST, and AM3 as a complex medium (pH 7.0) (Oxoid, Hampshire, United Kingdom), providing adequate growth and a broader distribution of MICs and MFCs (47, 58, 60).

### Fungicidal-activity testing.

After MIC determination, MFCs were determined by removing 10 μL from all wells displaying no visible growth and from the growth control (drug-free medium), followed by preparing 1:100 dilutions in spore suspension buffer (*n* = 100) (41, 61). Afterward, 100 μL was spread on SDA plates using a Whitley Automated Spiral Plater (model WASP 2, Don Whitley, Shipley, United Kingdom). SDA plates were incubated at 37°C for 48 h, and CFU/mL was counted. The MFC of AMB was defined as the lowest drug concentration which approximately killed 99% of the inoculum.

### Determination of tolerant phenotypes.

**(i) Definitions.** Due to the lack of clinical breakpoint (CBP) for AMB and A. terreus, categorization in the present study was adopted according to the CBP defined for AMB and A. fumigatus, which is ≤1 mg/L (62, 63). Isolates with AMB MICs of ≤1 mg/L were categorized as susceptible, and isolates with AMB MICs of >1 mg/L as resistant. Tolerance defines the ability of a phenotypical susceptible isolate (AMB MICs of ≤1 mg/L) to survive high AMB concentrations for a longer time than the susceptible control. Hence, the MDK99 of a tolerant strain is longer than the MDK99 of a susceptible one (33, 38, 39).

**Determination of the minimum duration of killing by time-kill assay**. Four isolates of A. terreus *sensu stricto* (s.s.) with different susceptibility profiles in a susceptible (≤1 mg/L) and resistant (>1 mg/L) range, which showed identical MICs in both RPMI 1640 and AM3 based on EUCAST method, were chosen for the time-kill assay: A. terreus 164 (S164, susceptible control, MIC = 0.5 mg/L; A. terreus s.s. 31 (T31), MIC = 1 mg/L; A. terreus s.s 81 (T81), MIC = 1 mg/L; and A. terreus s.s 134 (R134, resistant control), MIC = 4 mg/L. The time-kill analyses were performed as previously described with minor modifications using RPMI 1640 and AM3 (23). The time-kill assays were conducted in RPMI 1640 and AM3 using three concentrations of AMB (1×, 20×, and 40× MIC) and an untreated growth control. Freshly harvested spores of each isolate of A. terreus s.s. were prepared, and the inoculum of each isolate (1 × 10^5^ spores/mL) was added to 20 mL of each medium, RPMI 1640 and AM3. At different time points (0, 6, 12, 18, 24, 30, 36, 42, and 48 h), 100-μL aliquots were taken, diluted 100-fold in spore buffer, and 100 μL were cultured on SDA plates at 37°C (48h) for counting CFU/mL. The detection limit was defined to be 1 × 10^3^ CFU/mL. MDK99 was defined as the minimum time needed to reach a 99% reduction of the total number of CFU/mL from the initial inoculum at a specific concentration. Killing curves were constructed by plotting the log_10_ CFU/mL versus time over 48 h of each test condition against the control in Graph Pad Prism (version 8.0) software. Experiments were done three times and the mean values of three independent counts were used.

**Determination of spore germination rate.** Freshly harvested conidia from each of the four strains (S164, T81, T31, and R134) were washed three times with spore buffer and adjusted to 1 × 10^5^ spores/mL in RPMI 1640 and AM3. A 200-μL volume of each solution was placed in a 96-well plate and incubated at 37°C for 12 h. The germination rate was determined microscopically at each time by determining conidia that already had formed a germ-tube out of 100 randomly chosen conidia. Assays were carried out three times, and these three independent counts were used to calculate the percentage of germination.

### Assessing AMB efficacy against *A*. *terreus* isolates in *G*. *mellonella*.

To examine the relevance of the *in vitro* MDK99 results and the *in vivo* efficacy of AMB, four isolates (S164, T81, T31, and R134) exhibiting susceptible, tolerant, and resistant profiles were evaluated in the G. mellonella insect model as described previously (30). Briefly, groups of 20 larvae (0.3 to 0.4 g; SAGIP, Italy) were stored in wood shavings in the dark at 18°C for 24 h prior to the experiment. Three control groups were included: larvae injected with 20 μL sterile insect physiological saline (IPS; 150 mM NaCl, 5 mM KCl, 10 mM EDTA, and 30 mM sodium citrate in 0.1 M Tris-HCl [pH 6.9]), larvae that received 0.5 μg AMB, and untouched larvae. Larvae were infected with 1 × 10^7^ conidia/larva of each of the four isolates of A. terreus s.s. (S164, T31, T81, and R134), and injected with 0.5 μg AMB in a volume of 20 μL of IPS per larva at 2 h postinfection. The survival rate was monitored for up to 144 h at 37°C. Experiments were conducted in triplicate, and the data from all experiments (60 larvae in total) were combined to calculate the average survival rates determined every 24 h for a 144-h duration.

### Statistical analysis.

Survival rates of G. mellonella were determined using Kaplan-Meier survival curves and analyzed with the log-rank (Mantel-Cox) method. Furthermore, one-way analysis of variance (ANOVA) followed by Dunnett’s multiple-comparison test (for time-kill assay) and a paired *t* test (for germination rate determination) were used to determine statistical differences, using GraphPad Prism version 9.0.0 for Windows (GraphPad Software, San Diego, California USA, www.graphpad.com). MIC_50_, MIC_90_, MFC_50_, and MFC_90_ values were calculated using Microsoft Office Excel 2016. *P* values of <0.05 were considered significant.
